# Reduced spike specific T-cell responses in COVID-19 vaccinated subjects undergoing SARS-CoV-2 breakthrough infection

**DOI:** 10.3389/fimmu.2025.1657082

**Published:** 2025-09-05

**Authors:** Stefania Varchetta, Federica Sole Golfetto, Patrizia Bono, Annapaola Callegaro, Tanya Fabbris, Andrea Favalli, Mariacristina Crosti, Tullia Maria De Feo, Nathalie Iannotti, Giorgio Bozzi, Valeria Castelli, Bianca Mariani, Antonio Muscatello, Sergio Abrignani, Renata Grifantini, Alessandra Bandera, Andrea Lombardi

**Affiliations:** ^1^ Infectious Diseases Unit, Department of Internal Medicine, Fondazione IRCCS Ca’ Granda Ospedale Maggiore Policlinico, Milan, Italy; ^2^ Microbiology and Virology Unit, Foundation IRCCS Ca’ Granda Ospedale Maggiore Policlinico, Milan, Italy; ^3^ INGM, Istituto Nazionale Genetica Molecolare “Romeo ed Enrica Invernizzi”, Milan, Italy; ^4^ North Italy Transplant program (NITp). Transplant Coordination Unit, Fondazione IRCSS Ca’ Granda Ospedale Maggiore Policlinico, Milan, Italy; ^5^ Department of Clinical Sciences and Community Health, Università degli Studi di Milano, Milan, Italy; ^6^ Department of Pathophysiology and Transplantation, University of Milan, Milan, Italy

**Keywords:** SARS-CoV-2, vaccine, breakthrough infection, T cell immune responses, natural killercells, LAG-3

## Abstract

**Introduction:**

T-cell responses to SARS-CoV-2 remain largely preserved across variants despite waning neutralizing antibodies. However, T-cell immunity may vary with the host’s immune status, and data on T-cell responses in post-vaccine infections (PVI) are limited.

**Methods:**

We assessed Spike-specific T-cell responses in 32 vaccinated individuals, 16 of whom experienced PVI. Immune responses were evaluated at three time points: 1 month after the second vaccine dose (T1), 1 month after the booster dose (T2), and, in the PVI group, 1–3 months after the first positive nasal swab (T3). Additionally, we evaluated anti-spike antibody levels, T-cell exhaustion markers, and natural killer cell subsets, focusing on memory-like CD57^+^ NKG2C^+^ cells.

**Results:**

Subjects who developed PVI exhibited significantly reduced Spike-specific CD4 T-cell responses following the booster dose compared to vaccinated individuals who remained uninfected. This was accompanied by increased frequencies of LAG-3^+^ CD4^+^ and CD8^+^ T-cells. A positive correlation was observed between AIM^+^ CD4^+^ T-cells and NKG2C^+^ NK cells at T2 in PVI subjects. Following natural infection, T-cell responses were enhanced and associated with an expansion of NKG2C^+^ NK cells.

**Conclusions:**

Individuals experiencing PVI displayed impaired booster-induced CD4^+^ T-cell responses and increased expression of the immune checkpoint LAG-3. Natural infection restored and enhanced cellular immunity, particularly through the expansion of Spike-specific T-cells and memory NK cell populations. This study identifies an immune profile characterized by low spike-specific responses, which are associated with an increased susceptibility to breakthrough infections.

## 1 Introduction

Five years after the COVID-19 pandemic, significant data have been collected regarding the breadth and durability of the T-cell responses following SARS-CoV-2 infection or COVID-19 vaccination (1, 2). One significant discovery is the ability of T-cells to cross-recognize SARS-CoV-2 variants, including those with extensive mutations in the spike protein (3, 4). Indeed, despite the immune evasion observed in neutralizing antibody responses, T-cell responses have remained largely preserved across variants (5–8).

This preservation of T-cell functionality may explain the effectiveness of vaccines against severe disease, even when breakthrough infections occur with antigenically distinct variants (9). This cross-reactivity is particularly evident in CD4^+^ T-cells, which target conserved epitopes in the spike protein (10, 11). While CD8^+^ T-cell responses are more variable, these cells can adapt by generating *de novo* responses to mutated epitopes following breakthrough infections (12, 13). Tarke et al. demonstrated that over 80% of CD4^+^ and CD8^+^ T-cell epitopes are conserved across variants of concern, suggesting a robust cellular immune memory that is less susceptible to viral escape than humoral immunity (14).

Multiple studies have identified breakthrough infections as potentially beneficial immunological events that broaden the immune response beyond that achieved by vaccination alone (15, 16). These infections may function as natural boosters, especially against emerging variants not represented in the original vaccine formulations (17).

However, the precise immunological profile characterizing subjects who undergo breakthrough infections, particularly at the T-cell level, remains incompletely characterized.

Notably, Natural killer (NK) cells have been identified as essential components in the orchestration of vaccine-induced immune responses and are considered a promising target for enhancing vaccination strategies (18). These cells rapidly produce cytokines, such as IFN-γ, which can induce T-cell activation, promote dendritic cell maturation and the priming of virus-specific T-cells. In particular, increasing data support a critical role for “memory-like” NK cells in both the induction and the effector phases in response to vaccines (19–23). This subset of NK cells, identified in CMV-positive individuals, is characterized by the expression of the NKG2C^+^ activating receptor (19, 24). Memory-like NK cells may recognize peptide-HLA-E complex through binding to NKG2C (25). The therapeutic potential of these cells is recognized by their employment in many clinical trials for viral infections and cancer (23, 26–29).

In this study, we conducted a comprehensive analysis of NK and T-cell immunomodulation, including Spike-specific T-cell responses in 32 COVID-naïve individuals vaccinated with three doses of the original (Wuhan-Hu-1) mRNA or adenoviral vaccine. Among them, 16 experienced a breakthrough infection following booster dose during the December 2021-April 2022 period, when the Omicron sublineage BA.1 was the predominant SARS-CoV-2 variant in Italy (30, 31).

Our findings reveal unexpected dynamics in the CD4^+^ and CD8^+^ T-cell compartments following booster vaccination and breakthrough infection and provide essential insights into the complex interplay between vaccination and breakthrough infection in shaping T and NK-cell immunity against SARS-CoV-2.

## 2 Materials and methods

### 2.1 Study design and participants

Healthcare workers from Fondazione IRCCS Ca’ Granda Policlinico in Milan, Italy, were enrolled during the initial COVID-19 vaccination campaign. All participants received their first COVID-19 vaccination schedule with one of the following vaccines as primary regimen: BNT162b2 (Comirnaty, Pfizer–BioNTech), ChAdOx1-S (Vaxzevria, AstraZeneca–Oxford) or mRNA-1273 (Spikevax, Moderna–NIAID). Booster doses consisted of BNT162b2 (Comirnaty, Pfizer–BioNTech) or mRNA-1273 (Spikevax, Moderna–NIAID). **Table 1** shows the demographic and vaccination features of the subjects included in the study.

**Table 1:** Demographic data and vaccination schedule in controls and PVI subjects.

Demographic and vaccination features	CTRL (N=16)	PVI (N=16)	Total (N=32)
Age, median (range)	45.5 (25-64)	43.5 (26-58)	44.5 (25-64)
Female gender, n (%)	8 (50.0)	9 (56.2)	17 (53.1)
1° -2° vaccine dose			
BNT162b2 (Comirnaty, Pfizer–BioNTech) n (%)	7 (43.7)	3 (18.7)	10 (31.2)
ChAdOx1-S (Vaxzevria, AstraZeneca–Oxford n (%)	9 (56.2)	10 (62.5)	19 (59.3)
mRNA-1273 (Spikevax, Moderna–NIAID) n (%)	0	3 (18.7)	3 (9.3)
3° vaccine dose			
BNT162b2 (Comirnaty, Pfizer–BioNTech) n (%)	15 (93.7)	12 (75.0)	27 (84.3)
ChAdOx1-S (Vaxzevria, AstraZeneca–Oxford), n (%)	0	0	0
mRNA-1273 (Spikevax, Moderna–NIAID) n (%)	1 (6.2)	4 (25.0)	5 (15.6)
Days between booster dose and PVI; median(IQR)	–	54 (38 - 123)	–
Days between PVI and T3; median (IQR)	–	49 (28-62)	–

PVI, Post Vaccine Infection.

**Figure 1:**
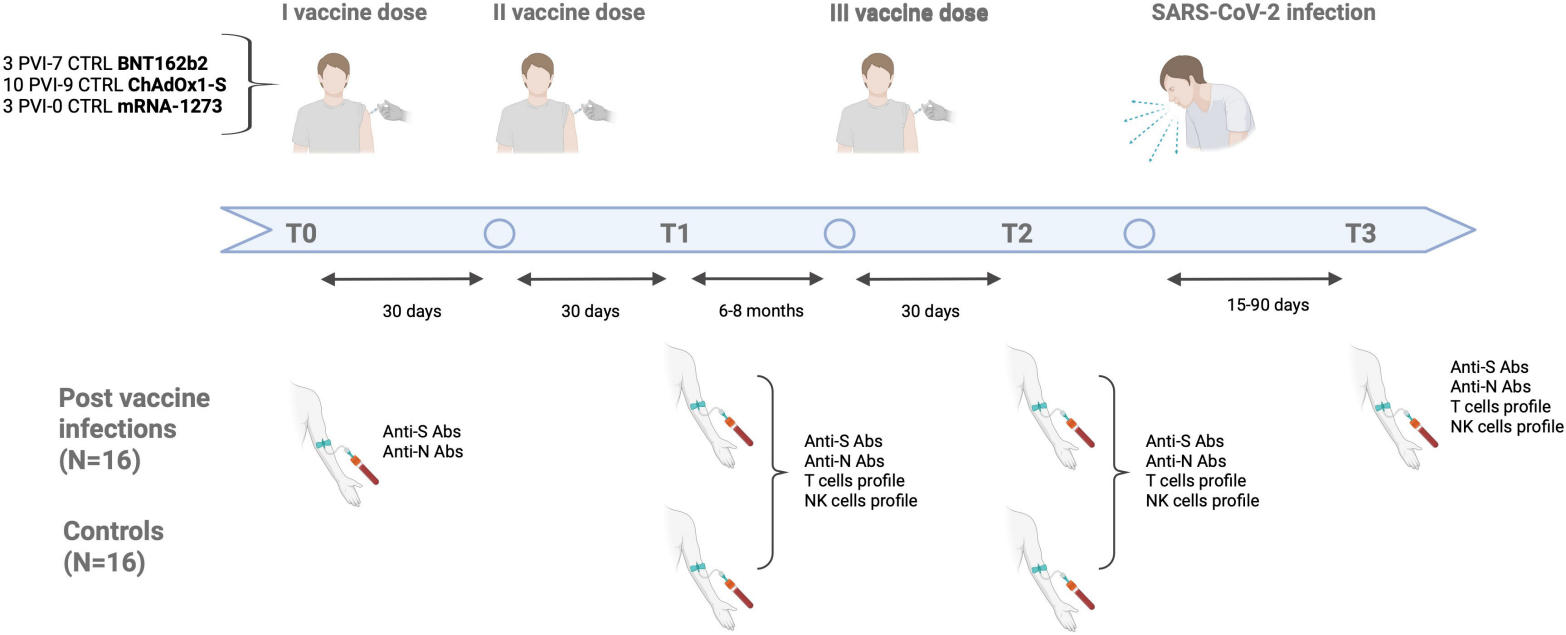
Time of vaccination and blood sampling scheme.

Among them, we identified 16 subjects with SARS-CoV-2 infection between 1–3 months after the third vaccine dose (post-vaccine infection, PVI) group. SARS-CoV-2 infection was identified through positive PCR on nasal swab, performed in all study participants reporting symptoms compatible with influenza-like illness. Sixteen control subjects of the same sex and similar age were also included in the study (CTRL group). We included in the survey only subjects who tested negative for SARS‐CoV‐2 anti-nucleocapsid IgG and provided informed consent for the study. Immune responses were examined in peripheral blood mononuclear cells (PBMC) collected at the following time points: 1 month after the second dose (T1), 1 month after the third dose (T2, booster) and 15–90 days following the first positive swab (only for IPV group) (T3). **Figure 1** shows time sampling and time of infection.

All subjects signed an informed consent. The study protocol adhered to the ethical guidelines outlined in the 1975 Declaration of Helsinki. It was approved by the Ethics Committee Lombardia 3 (document number 878, date of approval 18/03/2021).

#### 2.1.1 Isolation of peripheral blood mononuclear cells

Peripheral blood mononuclear cells (PBMC) were isolated by density gradient centrifugation (1.077 g/ml) using Ficoll-Plaque (Cedarlane, Burlington, Canada) according to the manufacturer’s instructions. Briefly, whole blood was diluted with an equal volume of phosphate-buffered saline (PBS) and layered on the Ficoll gradient. PBMC were isolated after centrifugation at 500 x g for 30 min at room temperature without brakes. PBMC were resuspended in PBS-EDTA and centrifuged at 400 × g for 10 min. Pellets were resuspended in PBS containing 2% FBS and washed by centrifugation at 250 × g for 10’ at room temperature. Cell numbers were determined by light microscopy count in a Burker chamber. Nonviable cells were identified by staining with trypan blue. Isolated PBMC were frozen in fetal bovine serum (FBS) with 10% dimethyl sulfoxide (DMSO) and stored in liquid nitrogen until use.

### 2.2 Peptide pools

A total of 53 synthetic 15-mer peptide pools, overlapping by 11 amino acid residues, covering the full-length Spike protein of the ancestral Wuhan strain (GenBank: MN_908947), were used as part of the PepTivator^®^ SARS-CoV-2 Prot_S Complete, premium grade (Miltenyi Biotec, Bergisch Gladbach, Germany). The peptide pools were dissolved following the manufacturer’s directions in sterile water. As a positive control, cells were stimulated with staphylococcal enterotoxin B (1 μg/mL, Sigma-Aldrich).

### 2.3 Activation-induced marker assay

Cryopreserved PBMC were thawed, resuspended in complete medium (RPMI 1640 containing 10% FBS, 1% L-glutamine, and 1% penicillin/streptomycin) and rested at 2 × 10^6^ cells for 2 hours at 37°C. Then, 1 × 10^6^ PBMC/well were plated in 96 well U-plates in complete medium in the presence/absence of 1μg/ml of Spike overlapping peptides. After 18 hours, PBMCs were washed and stained with CD3 BUV395 (clone UCHT1), CD56 RB613 (clone B159), CD4 APC (clone SK3), CD8 BUV737 (clone SK1), CD134 (OX40) RY586 (clone ACT35), CD137 (41BB) (RB744 (clone 4B4-1), CD69 BV480 (clone FN50), CD45RA RB545 (clone HI100), CD45RO BV786 (clone UCHL1), CCR7 BV711 (clone 2-L1-A), CD14 BUV496 (clone MφP9), CD19 BUV496 (clone SJ25C1) (BD Biosciences, CA, USA). A LIVE/DEAD^®^ Fixable Near-IR Dead Cell Stain Kit (Thermo Fisher Scientific, Waltham, MA, USA) was used to determine cell viability. After washing, cells were fixed with 2% paraformaldehyde in PBS and acquired with a FACS Symphony (BD Biosciences) flow cytometer. Spike-specific T cells were identified by activation-induced markers (AIM), measured as CD134^+^ CD137^+^ co-expression in CD4^+^ and as CD69^+^CD137^+^ co-expression in CD8^+^ T cell subsets (gating strategy shown in **Supplementary Figure 1**. The frequency of AIM^+^ T cells in Spike-stimulated samples was determined by subtracting the frequency of AIM^+^ T cells observed in the corresponding unstimulated (negative control) samples Three PVI subjects missed PBMC from timepoint 1. Staphylococcal enterotoxin B (SEB) 1 mg/ml) was used as positive control.

### 2.4 NK cell immunophenotype

Natural killer cell phenotype was assessed on thawed PBMC after resting for 2 hours at 37°C. A LIVE/DEAD^®^ Fixable Near-IR Dead Cell Stain Kit (Thermo Fisher Scientific) was used to determine cell viability. To identify NK cell subsets, the following antibodies were used: CD56 RB613 (clone B159), CD3 BUV563 (clone SK7), CD8 BUV737 (clone SK1), CD16 RY775 (clone 3G8), CD57 BUV395 (clone NK-1), CD69 BV480 (clone FN50), NKp46 BV786 (clone 9E2/NKp46), NKp30 BUV661 (clone p30-15), NKG2D BV711 (clone 1D11), CD127 RB744 (clone HIL-7R-M21), TIGIT BV650 (cloneTgMab-2), KLRG1 BV750 (clone Z7-205.rMAb), NKG2C BV421 (clone 134591) (BD Biosciences), NKG2AVio Bright B515 (clone REA1100) (Miltenyi Biotec, Bergisch Gladbach, Germany), Siglec-7 PE (clone QA79) ThermoFisher, MA, USA). After washing, cells were fixed with 2% paraformaldehyde in PBS and acquired with a FACSymphony A5 (BD Biosciences) flow cytometer. FlowJo software (v10.10) (BD Biosciences) was used to analyze data. An unsupervised approach was performed using the FlowJo plugin Uniform Manifold Approximation and Projection (UMAP) analysis on concatenated live NK cells. A total of 1260 NK cells for each individual were downsampled and concatenated before applying UMAP. Concatenated cells were clustered using FlowSom analysis. The cluster explorer plugin was used to identify cell clusters. In detail, 1260 NK cells were concatenated and analyzed with the FlowJo UMAP plugin. UMAP was run with the default settings (Euclidean distance function, nearest neighbors: 15 and minimum distance: 0.5). UMAP projections were obtained for concatenated cells from controls (n=16) and PVI subjects (n=16).

### 2.5 Antibody measurement

Elecsys Anti-SARS-CoV-2 S kit (Roche Diagnostics, Mannheim, Germany) was used to assess the development of total anti-Spike antibodies following SARS-CoV-2 vaccination. Precisely, this assay predominantly detects anti-RBD antibodies. Elecsys Anti-SARS-CoV-2 kit (Roche) was used to evaluate the development of total anti-N antibodies following SARS-CoV-2 infection. The threshold values were 1.0 Cut-Off Index (COI) for anti-N and 0.8 U/mL for anti-S.

### 2.6 Statistical analysis

Statistical analysis and graphical presentations were performed using GraphPad Software version 10.5.0 (GraphPad Software Inc, La Jolla, CA). Statistical differences between data within the same group were assessed by the non-parametric Friedman test followed by Dunn’s multiple comparisons or by the Wilcoxon matched-pairs signed-rank test. A mixed-effects model (REML) with Holm-Sidak’s multiple comparisons test was used, accounting for missing data. The Mann-Whitney U test was used to compare the differences between the two groups. The Shapiro-Wilk test was used to determine whether the data were normally distributed. The Fisher’s exact test was used to compare frequencies of responders to stimulation above the cut-off threshold (median values obtained after stimulation with the vehicle control for the AIM assay, 2 for the stimulation index).

## 3 Results

### 3.1 Study participants

This monocentric study recruited 32 COVID-19-naïve subjects who were vaccinated against COVID-19 at the Policlinico of Milan between January and December 2021. Seventeen subjects (53.1%) were female. The median age was 44.5 (range 25-64) years. The age of the control group ranged from 25 to 64 years, and the PVI group from 26 to 58 years. Only one subject, in the control group, was over 60. None of the subjects had comorbidities, except one control with obesity (**Table 2**). All patients received COVID-19 vaccination with a primary regimen of two vaccine doses followed by a third booster dose. The vaccines employed were BNT162b2, ChAdOx1-S or mRNA-1273. The adenoviral Chadox1-S vaccine was administered only as a primary regimen in 19 individuals and given in two doses separated by 4–6 weeks. The mRNA vaccines BNT162b2 and mRNA-1273 were used as primary regimens in 13 subjects and for boosting in all participants. When used as a primary regimen, these vaccines required two doses separated by 4–6 weeks. Among the 16 subjects with ascertained SARS-CoV-2 infection, none required hospitalization or oxygen supplementation with all belonging to WHO ordinal clinical severity scale 1 and 2. **Figure 1** shows time sampling and time of infection.

**Table 2:** Individual demographic characteristics, comorbidities and vaccine type.

Subject ID	Sex	Age (years)	Comorbidities	Vaccine type (1st/2nd dose)	Vaccine type (3rd dose)
CTRL 0004	M	32	None	BNT162b2	BNT162b2
CTRL 0009	F	64	None	BNT162b2	BNT162b2
CTRL 0011	F	53	None	BNT162b2	BNT162b2
CTRL 0012	F	45	Obesity	BNT162b2	BNT162b2
CTRL 0028	F	59	None	BNT162b2	BNT162b2
CTRL 0029	M	58	None	BNT162b2	BNT162b2
CTRL 0034	F	57	None	BNT162b2	BNT162b2
CTRL 0037	F	30	None	ChAdOx1 S	BNT162b2
CTRL 0040	F	51	None	ChAdOx1 S	BNT162b2
CTRL 0043	M	47	None	ChAdOx1 S	BNT162b2
CTRL 0052	M	46	None	ChAdOx1 S	BNT162b2
CTRL 0053	F	37	None	ChAdOx1 S	BNT162b2
CTRL 0068	M	25	None	ChAdOx1 S	mRNA - 1273
CTRL 0069	M	25	None	ChAdOx1 S	BNT162b2
CTRL 0072	M	33	None	ChAdOx1 S	BNT162b2
CTRL 0077	M	40	None	ChAdOx1 S	BNT162b2
PVI 0013	F	43	None	BNT162b2	BNT162b2
PVI 0030	M	58	None	BNT162b2	BNT162b2
PVI 0032	F	56	None	BNT162b2	BNT162b2
PVI 0035	F	52	None	ChAdOx1 S	BNT162b2
PVI 0039	M	38	None	ChAdOx1 S	BNT162b2
PVI 0044	M	50	None	ChAdOx1 S	BNT162b2
PVI 0055	M	38	None	ChAdOx1 S	BNT162b2
PVI 0059	M	47	None	ChAdOx1 S	BNT162b2
PVI 0065	F	30	None	ChAdOx1 S	BNT162b2
PVI 0076	M	31	None	ChAdOx1 S	mRNA - 1273
PVI 0078	F	33	None	ChAdOx1 S	BNT162b2
PVI 0084	F	35	None	ChAdOx1 S	BNT162b2
PVI 0087	M	26	None	ChAdOx1 S	BNT162b2
PVI 0127	F	58	None	mRNA - 1273	mRNA - 1273
PVI 0251	F	44	None	mRNA - 1273	mRNA - 1273
PVI 0264	F	46	None	mRNA - 1273	mRNA - 1273

### 3.2 Anti-spike and anti-nucleocapsid antibodies

**Figure 2:**
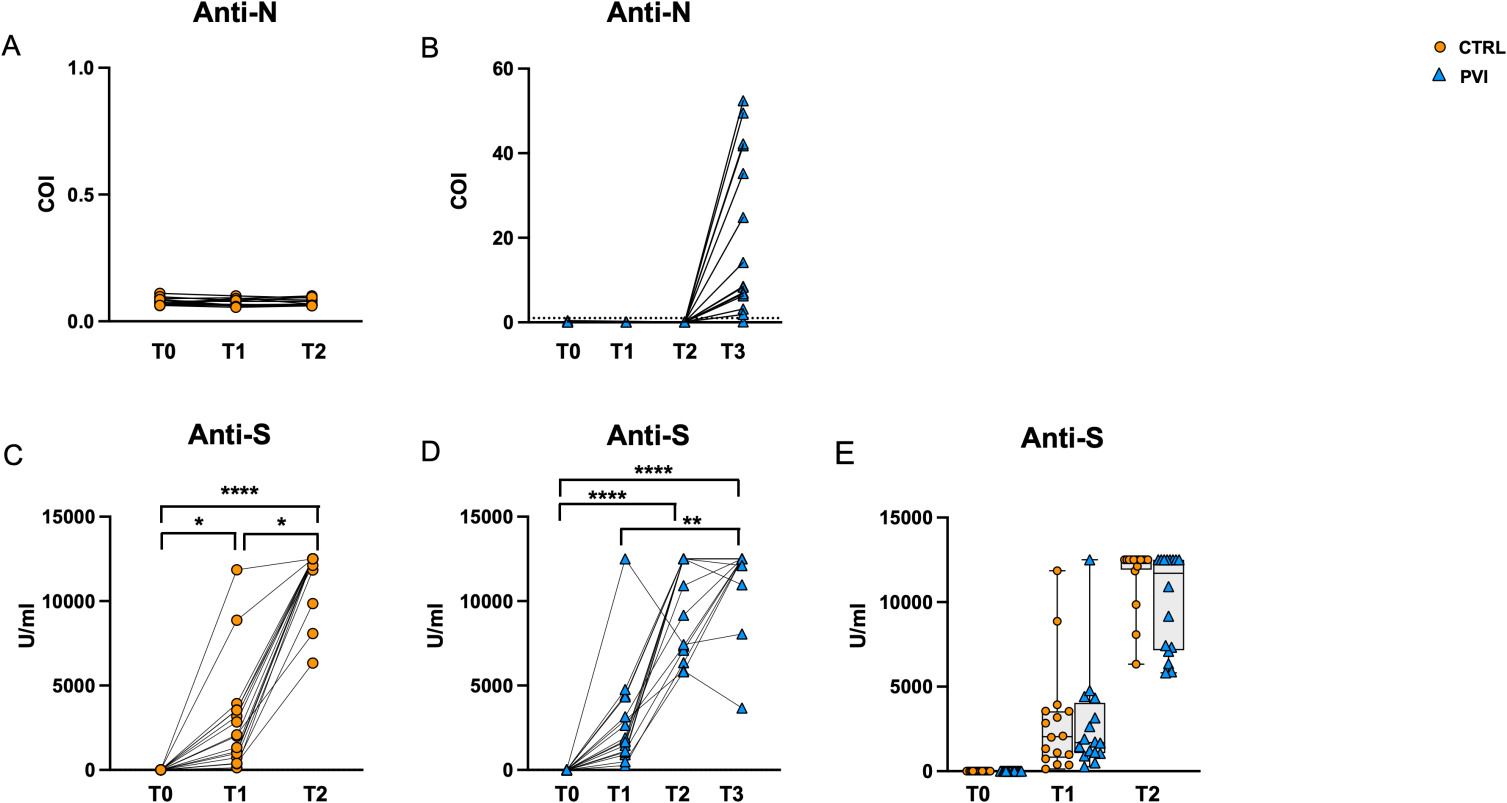
Antibody secretion following vaccination. Anti-N IgG tested negative in controls **(A)** and PVI **(B)** subjects at T0, T1 and T2. All PVI subjects except one developed anti-N antibodies following natural infection at T3 **(B)**. Anti-S antibodies were significantly increased among timepoints in control- **(C)** as well as IPV subjects **(D)**. Comparable anti-S antibodies were observed between IPVs (blue triangle) and CTRLs (orange circle) during vaccine timepoint follow-up **(E)**. Anti-N, anti nucleocapsid antibodies; anti-S, anti-Spike antibodies; COI, Cut-Off Index. The one-way Friedman with Dunn’s multiple comparison test was used to compare data within the same group. The non-parametric Mann–Whitney U test was used to assess statistical differences between groups. *p < 0.05; **p<0.01; ****p<0.0001.

All subjects were tested for serum levels of anti-N and anti-Spike antibodies at T0 (before vaccination) and at the subsequent timepoints (T1, T2 and T3) (**Figure 2**).

All sera were negative for anti-N antibodies at T0, T1 and T2, confirming the naïve status of all individuals during the study (**Figures 2A, B**). All PVI subjects except one developed anti-N antibodies at T3 following natural infection. However, there was substantial variation in anti-N antibody levels, ranging from 0 to 52 Cut-Off Index (COI) levels (**Figure 2B**). Anti-Spike antibodies were significantly increased along time points in both groups (**Figures 2C, D**), and their level was comparable in the two groups at all time points (**Figure 2E**).

### 3.3 T-cell maturation subsets

We investigated the immunophenotypic dynamics of CD4^+^ and CD8^+^during vaccine follow-up using multiparametric flow cytometry. The analysis of CD4^+^ T-cell maturation subsets revealed a consistent decline in naïve cells at T2 compared to T1 across both study groups. In contrast, central memory (CM) CD4^+^ T-cells increased significantly in the control group, with a similar trend in PVI subjects (p = 0.054). A trend toward a significant reduction of the frequency of CM CD4 T-cells was observed at T2 in PVI compared with controls (p= 0.08) (**Supplementary Figures 2A, B**). Notably, the PVI group exhibited a significant increase in effector memory (EM) and terminally differentiated effector memory T cells re-expressing CD45RA (EMRA) CD4^+^ T-cells at T3 (**Supplementary Figures 2C, D**).

Among CD8^+^ T-cells, controls exhibited a marked decrease in naïve and EMRA subsets at T2 (**Supplementary Figure 2D**), as well as a significant expansion of central memory cells (**Supplementary Figure 2E**). A considerable reduction of EMRA CD8 T-cells was present at T2 in the control group only (**Supplementary Figure 2G**), while no differences were observed among EM CD8 T-cells **Supplementary Figure 2F**).

### 3.4 COVID-19 vaccination was unable to induce an effective CD4^+^ and CD8^+^ T-cell response following the booster dose

**Figure 3:**
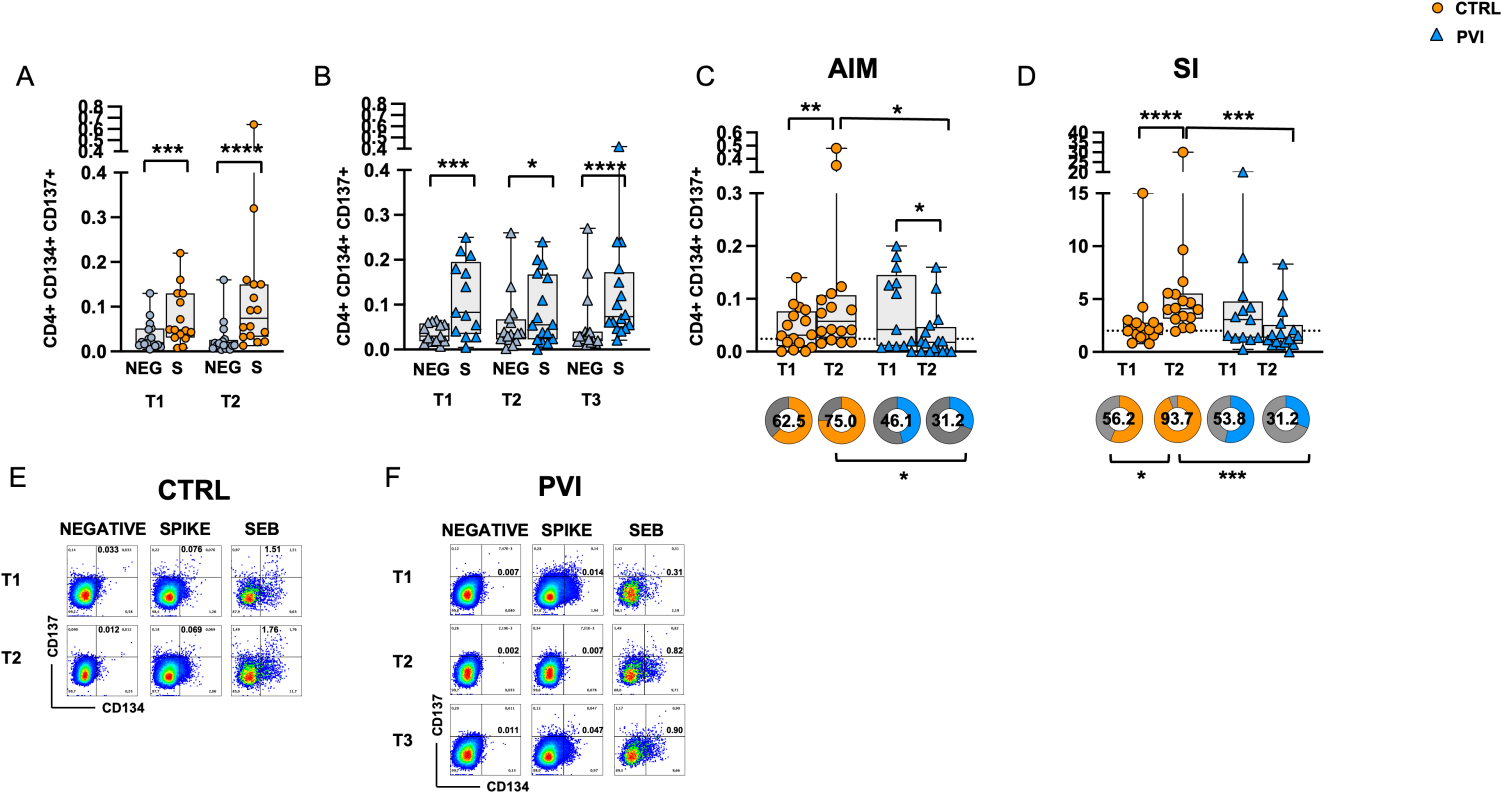
Expression of Activation-Induced Markers (AIMs) in CD4^+^ T-cells following stimulation with Spike overlapping peptides. Frequency of AIM-expressing CD4^+^ T-cells in unstimulated cells (grey symbol) or following stimulation with Spike (orange symbol) overlapping peptides in **(A)** the control group (circles) and **(B)** in the PVI group (triangles). **(C)** Spike-reactive CD4^+^ T-cells following stimulation with Spike overlapping peptides in controls and PVI subjects after subtraction of the negative control. Donut graph shows the frequency of subjects responding above the threshold in the two groups. **(D)** Stimulation Index in the control group and in the PVI group. Donut graph shows the frequency of subjects responding 2-fold above the stimulation index. Representative AIM dot plots from **(E)** one control and **(F)** one PVI subject. SI—Stimulation Index; SEB- Staphylococcal enterotoxin **(B)** Statistical differences between data within the same group were assessed by the Wilcoxon matched-pairs signed-rank test. Statistical differences between groups were assessed by the non-parametric Mann–Whitney U test. Fisher’s exact test was used to compare proportions. *p < 0.05; **p<0.01; ***p < 0.001; ****p<0.0001.

To assess Spike-specific recall responses, AIM analysis was performed on T-cells following stimulation with overlapping peptides (OP) spanning the Spike protein. Co-expression of OX40 (CD134) and 4-1BB (CD137) was used to identify Spike-specific CD4^+^ T-cell activation. CD4^+^ T-cells exhibited significantly increased activation following OP stimulation compared to the negative control (unstimulated cells) at all time points in both groups (**Figures 3A, B**). After background subtraction of AIM responses observed in the negative controls, the control group showed a significant increase in the frequencies of AIM^+^ CD4^+^ T-cells at T2 compared to T1. At T1, the comparison between the two groups showed similar frequencies of Spike-specific CD4^+^ T-cells. However, at T2, the PVI group exhibited a reduced frequency of AIM^+^ CD4 T-cells compared to controls (**Figure 3C**); furthermore, the proportion of subjects with responses above the threshold (defined as the median of negative control values) was also significantly lower in the PVI group at T2.

Additionally, the stimulation index (SI), calculated as the ratio of AIM^+^ CD4^+^ T-cell frequencies in stimulated versus unstimulated cells, was significantly increased at T2 compared to T1 in the control group only. Notably, at T2, the IPV group had a considerably lower SI compared to controls (**Figure 3D**). Consistently, the proportion of subjects with a ≥2-fold SI response was significantly reduced in the PVI group at T2 (**Figure 3D**). Representative CD4+ AIM dot plots are shown in **Figures 3E, F**.

**Figure 4:**
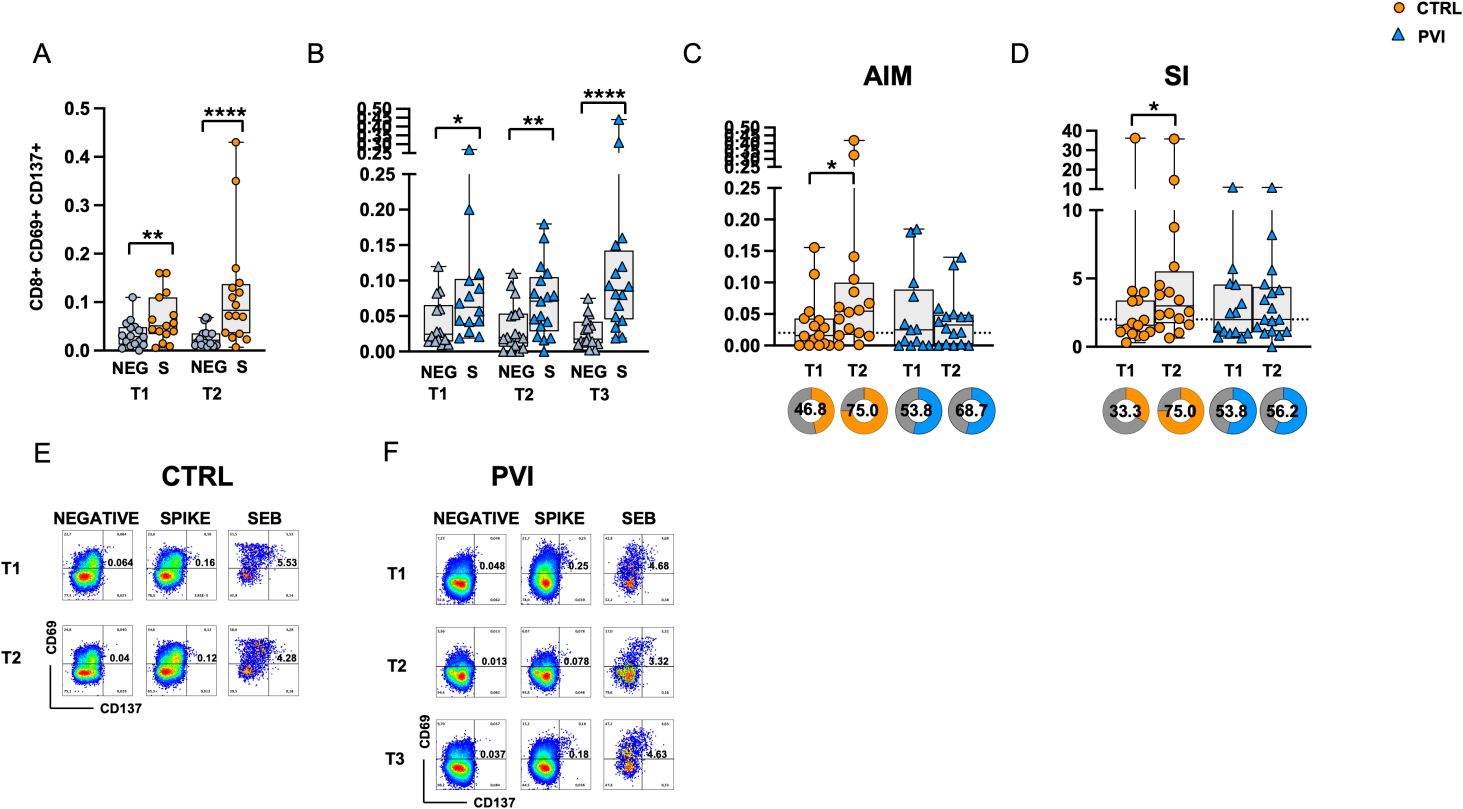
Expression of Activation-Induced Markers (AIMs) in CD8^+^ T-cells following stimulation with Spike overlapping peptides. Frequency of AIM-expressing CD8^+^ T-cells in unstimulated cells (grey symbol) or following stimulation with Spike (orange symbol) overlapping peptides in **(A)** the control group (circles) and **(B)** in the PVI group (triangles). **(C)** Spike-reactive CD8^+^ T-cells following stimulation with Spike overlapping peptides in controls and PVI subjects after subtraction of the negative control. Donut graph shows the frequency of subjects responding above the threshold in the two groups. **(D)** Stimulation index in the control group and in the PVI group. Donut graph shows the frequency of subjects responding 2-fold above the stimulation index. Representative AIM dot plots from **(E)** one control and **(F)** one PVI subject. SI—Stimulation Index; SEB- Staphylococcal enterotoxin **(B)** Statistical differences between data within the same group were assessed by the Wilcoxon matched-pairs signed-rank test. Statistical differences between groups were assessed by the non-parametric Mann–Whitney U test. Fisher’s exact test was used to compare proportions. *p < 0.05; **p<0.01; ****p<0.0001.

Spike-specific CD8^+^ T-cell responses were assessed through CD69 and CD137 co-expression analysis. Following stimulation with Spike OP, CD8^+^ T-cell responses were significantly increased compared to the negative control at all time points in both groups. (**Figures 4A, B**). No differences in antigen-specific AIM responses were observed between the two groups after subtracting the AIM responses in negative control (**Figures 4C**). However, a significantly increased frequency of Spike-specific CD8^+^ T-cells was observed at T2 compared to T1 in the control group only. Similarly, a significant increase in the SI was observed at T2 compared to T1 in the control group only (**Figure 4D**). No significant differences were observed among the frequency of subjects responding above the AIM or SI threshold (**Figures 4C, D**). Representative CD8+ AIM dot plots are shown in **Figures 4E, F**.

### 3.5 Natural infection restores spike-specific T-cell immunity

**Figure 5:**
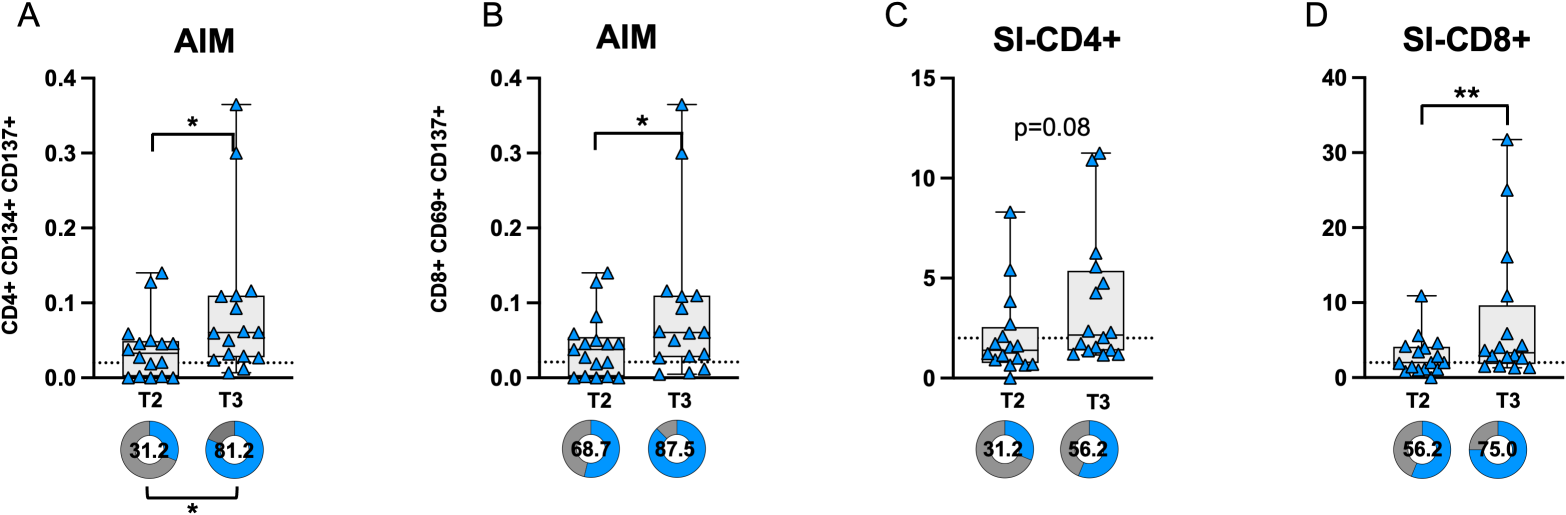
AIM expression in CD4 **(A)** and CD8 **(B)** T-cells before (T2) and after (T3) breakthrough infection in PVI subjects. Donut graph shows the frequency of subjects responding above the threshold in the two groups. Stimulation index in CD4 **(C)** and CD8+ **(D)** T-cells. Donut graph shows the frequency of subjects responding 2-fold above the stimulation index. SI—Stimulation Index; Statistical differences were assessed by the Wilcoxon matched-pairs signed-rank test. Fisher’s exact test was used to compare proportions. *p < 0.05; **p<0.01.

Comparison of CD4 and CD8 T-cell responses before (T2) and after (T3) COVID breakthrough infection revealed a significant increase in AIM expression in both CD4^+^ and CD8^+^ T-cells at T3 compared to T2 (**Figures 5A, B**). The proportion of subjects with AIM^+^ CD4^+^ T-cell responses above the threshold (defined by the median of negative controls) was significantly higher at T3 compared to T2. Additionally, the SI was significantly elevated in CD8^+^ T-cells at T3, while a trend toward significance was observed in CD4^+^ T-cells (p = 0.083) (**Figures 5C, D**).

### 3.6 Increased LAG-3 expression in CD4 and CD8 T-cells

**Figure 6:**
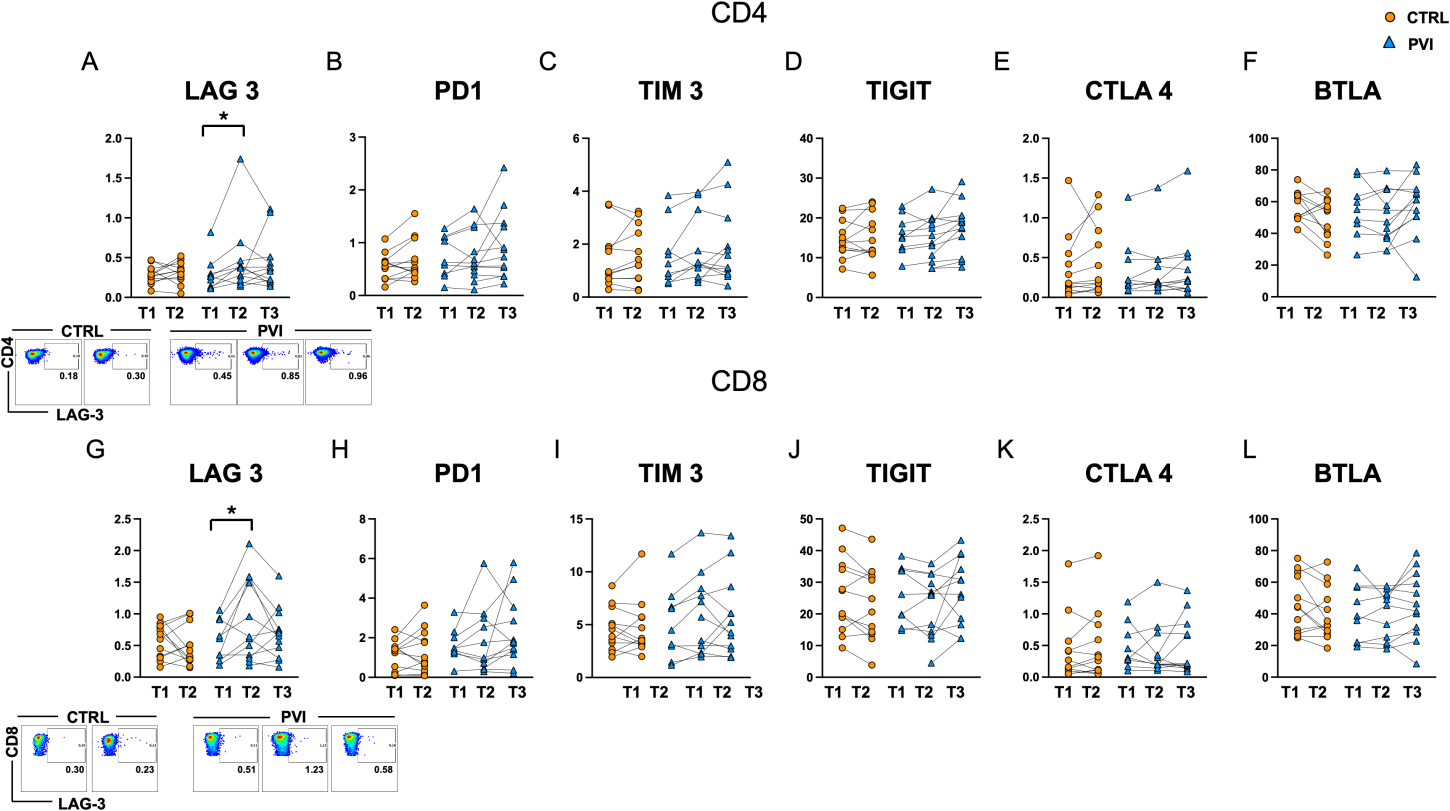
Levels of exhaustion markers in CD4^+^ **(A-F)** and CD8^+^ **(G-L)** T-cells in controls (orange circles) and PVI subjects (blue triangles). LAG-3 representative dot plots are shown for CD4^+^ (upper panel) and CD8^+^ (lower panel) cells. Statistical differences were assessed by the Wilcoxon matched-pairs signed-rank test and Holm-Šídák’s multiple comparisons test. *p < 0.05.

To investigate the potential involvement of T-cell dysfunction in the reduced CD4^+^ T-cell response, we assessed the expression of several co-inhibitory receptors commonly associated with exhaustion, including LAG-3, PD-1, TIM-3, TIGIT, CTLA-4, and BTLA on CD4^+^ and CD8^+^ T-cells. A significant increase in the proportion of LAG-3-positive CD4^+^ and CD8^+^ T-cells was observed in the PVI group at T2, following the booster dose, compared to T1 (**Figures 6A, G**).

Upregulation of exhaustion-associated markers can occur as part of the normal T-cell activation process, for example after vaccination. However, the level of expression is generally expected to remain comparable across successive doses and between individuals mounting similarly effective responses. In this context, the increase in LAG-3 expression, particularly among CD8+ T-cells, was more pronounced in individuals who subsequently experienced a breakthrough infection. This could suggest a qualitative difference in the immune response, potentially reflecting a state of early or functional exhaustion. However, given that these markers can also reflect recent activation, further functional assays would be necessary to distinguish between activation-associated expression and true exhaustion. No significant differences were detected for the other exhaustion markers analyzed (**Figure 6**).

### 3.7 Memory-like NK cell expansion in PVI

**Figure 7:**
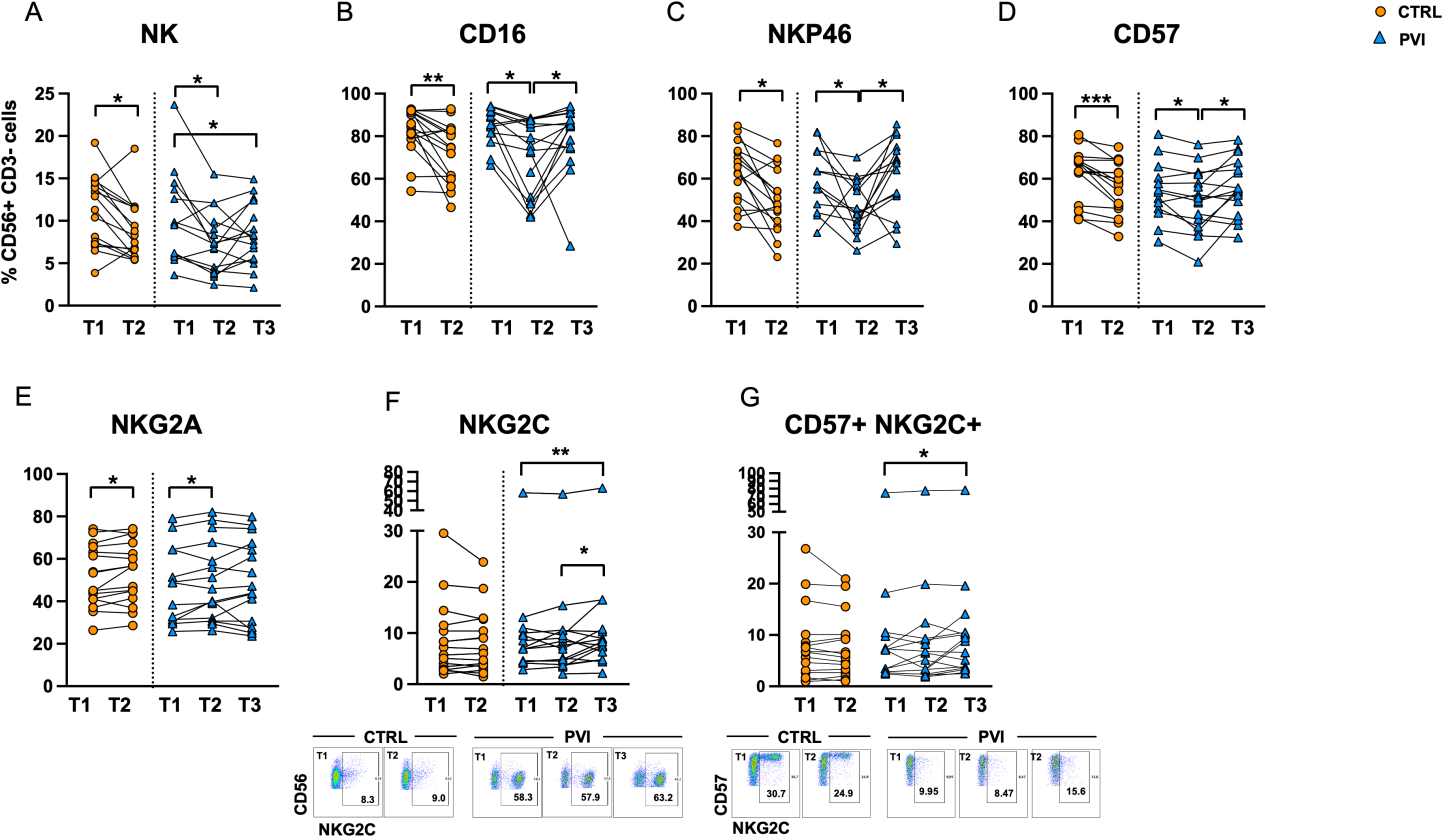
NK cell frequency in controls (orange circles) and PVI subjects (blue triangles) **(A)**. Proportion of NK cells expressing CD16^+^ **(B)**, NKp46^+^ **(C)**, CD57 **(D)**, NKG2A **(E)**, NKG2C **(F)** and CD57^+^ NKG2C^+^ memory-like NK cells **(G)** in controls (orange circles) and PVI (blue triangles) subjects. NKG2C^+^ and CD57^+^ NKG2C^+^ representative dot plots are shown. Statistical differences were assessed by the Wilcoxon matched-pairs signed-rank test and Holm-Šídák’s multiple comparisons test. *p < 0.05; **p<0.01; ***p < 0.001.

Fifteen different antibodies (listed in Materials and Methods) were used to analyze the immunophenotype of NK cells by flow cytometry. The frequency of total CD56^+^ CD3^-^ NK cells was significantly reduced at T2 in both PVI and controls. Additionally, a significant decrease was observed at T3 compared to T1 in the PVI group (**Figure 7A**). The immune profiling of NK cells revealed a marked reduction in the expression of the cell activation marker CD16 in both groups at T2, followed by a significant increase at T3 in PVI subjects (**Figure 7B**). Similarly, the frequencies of the activating receptor NKp46 and the maturity marker CD57 were reduced at T2 compared to T1 in both groups, with both markers showing a significant increase at T3 in the PVI group (**Figures 7C, D**). The frequency of the inhibitory receptor NKG2A increased at T2 compared to T1 in both groups (**Figure 7E**). Notably, NKG2C^+^ and CD57^+^ NKG2C^+^ memory-like NK cells were significantly increased at T3 in PVI subjects (**Figures 7F, G**). Unsupervised analysis identified 12 distinct NK cell clusters; among these, Cluster 2 showed increased frequency in the PVI group and exhibited a phenotype consistent with memory-like NK cells (CD57^+^, NKG2C^+^, NKG2A^-^, Siglec-7^low^, NKp46^low^) (**Supplementary Figure 3**). Interestingly, a comparison of Cluster 2 between the two groups revealed a distinct receptor expression pattern associated with PVI, characterized by a reduced proportion of CD56^+^CD8^+^ NK cells and increased expression of NKp46 (**Supplementary Figure 3E**).

**Figure 8:**
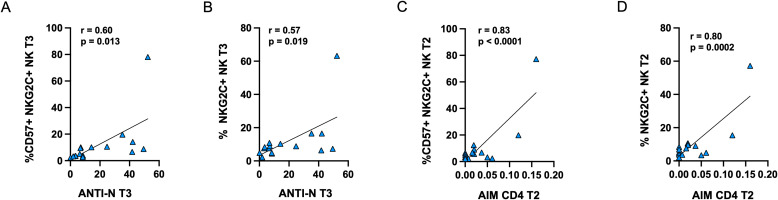
Positive correlations between anti-N antibodies and the frequency of CD57^+^NKG2C^+^ **(A)** or NKG2C **(B)** memory-like NK cells in PVI subjects at T3. Positive correlations between the frequency of AIM^+^ CD4^+^ T-cells and CD57^+^NKG2C^+^ **(C)** or NKG2C **(D)** memory-like NK cells in PVI subjects at T2. Correlations between variables were analyzed by Pearson’s rank correlation coefficient.

Furthermore, the frequencies of memory-like CD57^+^NKG2C^+^ and NKG2C^+^ NK cells at T3 were positively correlated with anti-N antibody levels at the same time point (**Figures 8A, B**). Additionally, in PVI subjects only, the proportion of memory-like NK cells at T2 was positively correlated with the frequency of AIM^+^CD4^+^ T-cells at the corresponding time point (**Figures 8C, D**).

## 4 Discussion

This study provides a comprehensive analysis of Spike-specific T-cell responses in a cohort of SARS-CoV-2-unexposed subjects who experienced breakthrough infection following the booster dose of the COVID-19 vaccine. Notably, in the period following the third vaccine dose, individuals who later developed PVI exhibited a distinct immunological profile characterized by significantly reduced Spike-specific CD4^+^ T-cell responses. This impaired cellular immunity was restored after natural infection, as demonstrated by enhanced antigen-specific CD4^+^ and CD8^+^ T-cell activation post-infection.

Our findings are consistent with a previous study, which reported reduced spike-specific T-cell responses in individuals who subsequently developed COVID-19 compared to those who remained uninfected (32). Another study showed that older adults who later experienced PVI displayed significantly lower vaccine-induced spike-specific CD4^+^ and CD8^+^ T-cell responses compared to those who remained uninfected; however, in contrast to our data, they did not observe any significant differences in the frequency of spike-specific T-cells among younger population suggesting that host-related factors such as age and immune competence may influence susceptibility to PVI (33).

Similarly, Rovida et al. reported no significant differences in T-cell responses between PVI and non-PVI individuals after two doses of the BNT162b2 vaccine (34). However, it is essential to note that immune responses were assessed during acute infection (within 48 hours of diagnosis), potentially masking pre-infection differences due to infection-induced immune activation. This methodological difference may explain the discordant results and emphasizes the importance of timing in immunological assessments.

Interestingly, we found that the reduction in T-cell responses after booster vaccination in PVI subjects was accompanied by increased expression of the immune checkpoint receptor LAG-3, particularly in CD8^+^ T cells. LAG-3 is typically associated with T cell exhaustion, particularly in the context of chronic infection or persistent antigen exposure. Indeed, prior studies have linked high LAG-3 expression to both mild and severe COVID-19 cases (35) and dysfunctional antiviral responses (36). However, co-inhibitory markers are also known to be transiently upregulated following T-cell activation. Therefore, the increased LAG-3 expression observed in individuals who later experienced breakthrough infection may indicate an altered immune profile, potentially indicative of early dysfunction, or reflect recent activation. Further functional studies are needed to better understand its significance.

In line with this, our data show a trend towards decreased central memory CD4^+^ T-cells in PVI subjects. Central memory T-cells have a prominent role in peptide-induced recall responses and are enriched following effective vaccination or infection (2, 37). The reduction of the CM T-cell pool may contribute to weaker T-cell recall responses to Spike peptides post-booster, as confirmed by lower AIM^+^ CD4^+^ T-cell frequencies and stimulation index in the PVI group.

In contrast to T-cells, the anti-Spike antibody levels were comparable between groups at all time points, suggesting that antibody titers alone may not predict protection against PVI. These findings are supported by previous work demonstrating that T-cell immunity is critical for long-term protection and disease control (38).

The evaluation of NK cell immunophenotype during vaccination revealed modulation within the memory-like NK cell compartment following vaccination and infection, with a significant increase in NKG2C^+^ NK cells post-infection in PVI individuals. These cells have been previously associated with robust antiviral responses in convalescent individuals (26) and have been shown to improve outcomes in SARS-CoV-2 infection. Indeed, the deletion of the NKG2C receptor has been associated with the development of severe COVID‐19 (39). Moreover, recent data have shown that memory-like natural killer cells exhibit protective activity against lung invasion during SARS-CoV-2 infection (40). Emerging evidence suggests that memory-like NKG2C^+^ NK cells play a crucial role in shaping effective vaccine responses, as they contribute to orchestrating T-cell immunity following COVID-19 vaccination, underscoring their importance in the development of vaccine-induced immunity (40).

A positive correlation between memory-like NK cell frequencies and both anti-N antibodies and AIM^+^ CD4^+^ T-cells was observed, suggesting a coordinated innate and adaptive antiviral immune activation. This finding aligns with previous reports showing that cooperation between innate lymphoid cells and vaccine-induced antibodies contributes to the regulation of vaccine-elicited T-cell responses, further supporting the idea of integrated crosstalk between innate and adaptive immunity during antiviral responses (41, 42).

Finally, in agreement with previous studies (15, 43, 44) our results confirm that natural infection boosts cellular immunity more robustly than vaccination alone, likely due to broader epitope exposure and higher antigenic load.

This study has several limitations. First, the small sample size limits the statistical power and applicability of the findings, particularly for subgroup comparisons. Second, while we assessed antigen-specific T-cell activation using AIM assays, we did not evaluate functional parameters such as cytokine production, which would provide deeper insights into the quality and breadth of the T-cell response. Third, immune responses were not tested against specific SARS-CoV-2 variants, which could have influenced susceptibility to breakthrough infection. Moreover, all the PVI included were of limited severity, not requiring hospitalization or oxygen supplementation. It cannot be ruled out that in more severe clinical manifestations the difference detected in our study can be more pronounced. Additionally, the study focused on circulating immune cells and did not assess mucosal immunity, a key component of protection against respiratory viruses. The use of different vaccine types among participants introduces heterogeneity, and potential confounders. Although the majority received either BNT162b2 or ChAdOx1-S for the initial two doses, a small subset received mRNA-1273. Similarly, the third dose included both BNT162b2 and mRNA-1273, with different distributions between the PVI and control groups. Given known differences in immunogenicity between vaccine platforms and mRNA dose, this variation could influence immune responses. Due to the limited sample size, stratified subgroup analyses were not feasible; thus, this heterogeneity should be considered when interpreting the findings. Finally, although multiple time points were analyzed, some immune dynamics may have been missed due to the limited sampling frequency, particularly after infection.

In summary, our study provides evidence that COVID-naïve, vaccinated individuals who later experience breakthrough infection display impaired Spike-specific CD4^+^ T-cell responses after the third vaccine dose, despite comparable antibody levels. This reduced cellular response may contribute to increased susceptibility to SARS-CoV-2 infection. Following natural infection, both T-cell and NK cell responses were restored or enhanced, indicating that natural exposure can overcome initial vaccine-induced immune limitations.

These findings underscore the importance of evaluating cellular immunity to predict vaccine efficacy and the risk of breakthrough infections. Moreover, they emphasize the need for additional research into host factors, including LAG-3, and the potential role of memory-like NK cells as biomarkers of immune competence.

## Data Availability

The raw data supporting the conclusions of this article will be made available by the authors, without undue reservation.
