# Supplementary figures and images for: Reduced spike specific T-cell responses in COVID-19 vaccinated subjects undergoing SARS-CoV-2 breakthrough infection
Source: Front Immunol. 2025 Sep 5;16:1657082. doi: 10.3389/fimmu.2025.1657082 (PMC12446237; doi:10.3389/fimmu.2025.1657082)

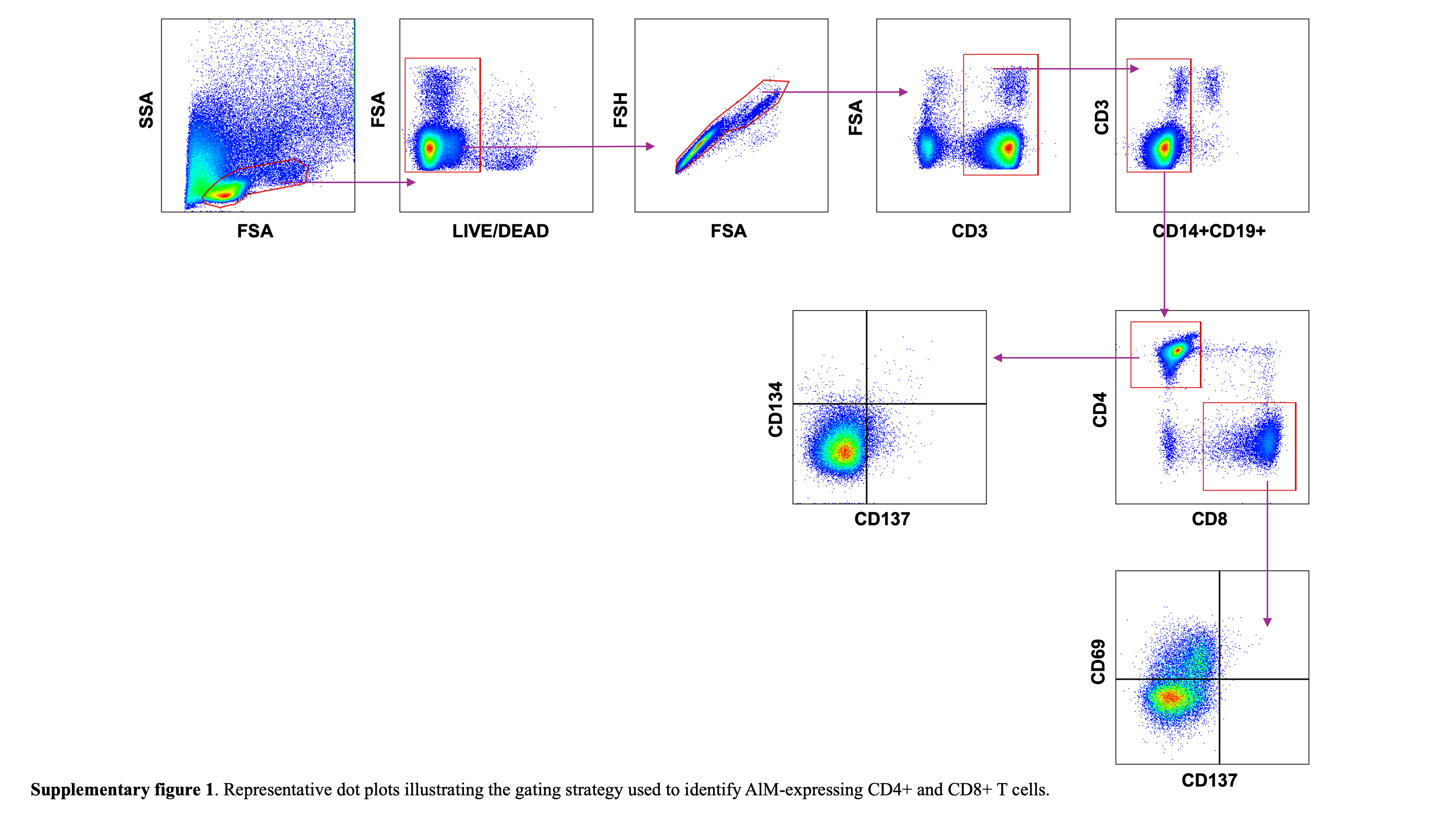

Supplement: Supplementary file 1 [file Image1.tiff]

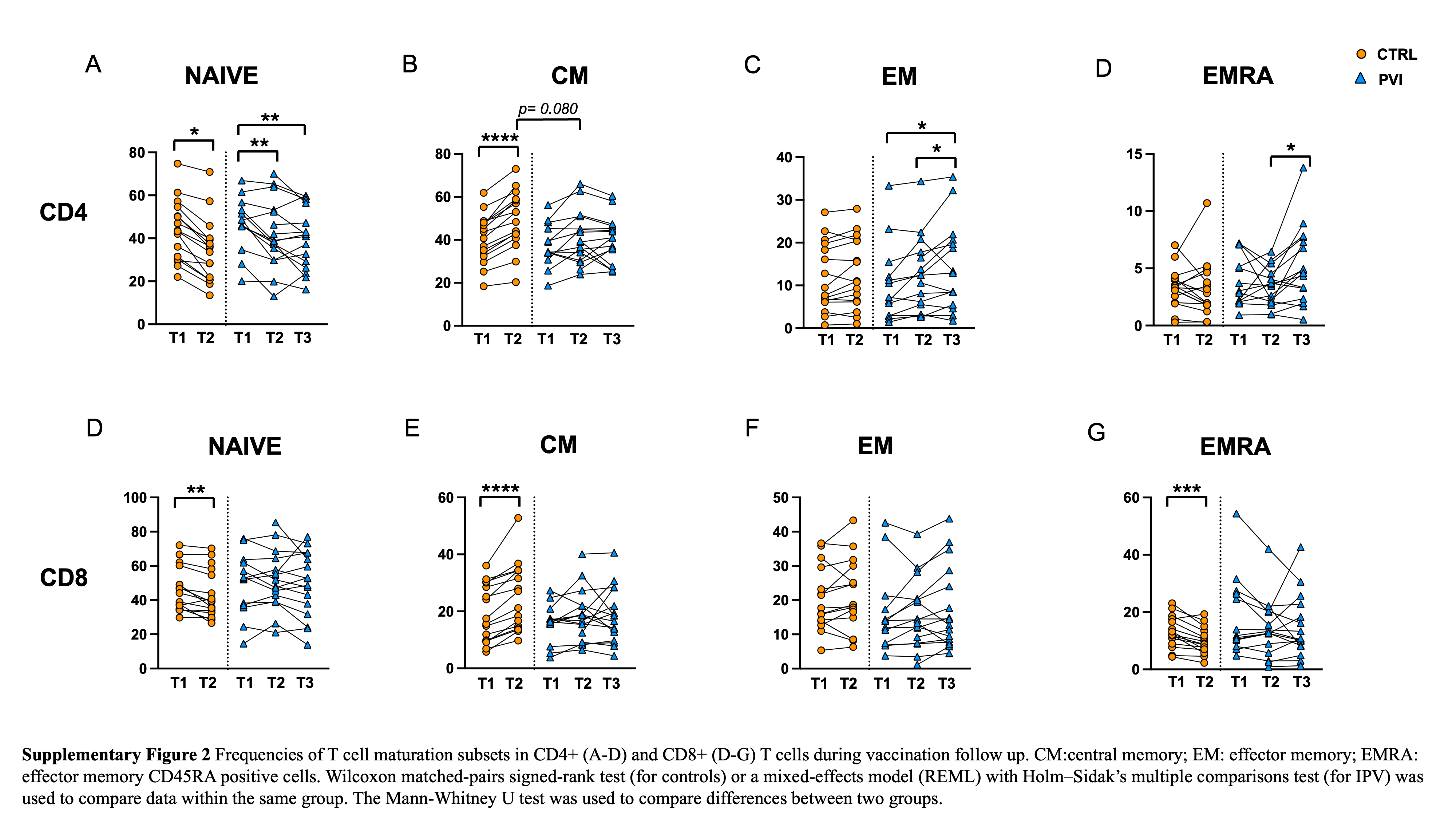

Supplement: Supplementary file 2 [file Image2.tiff]

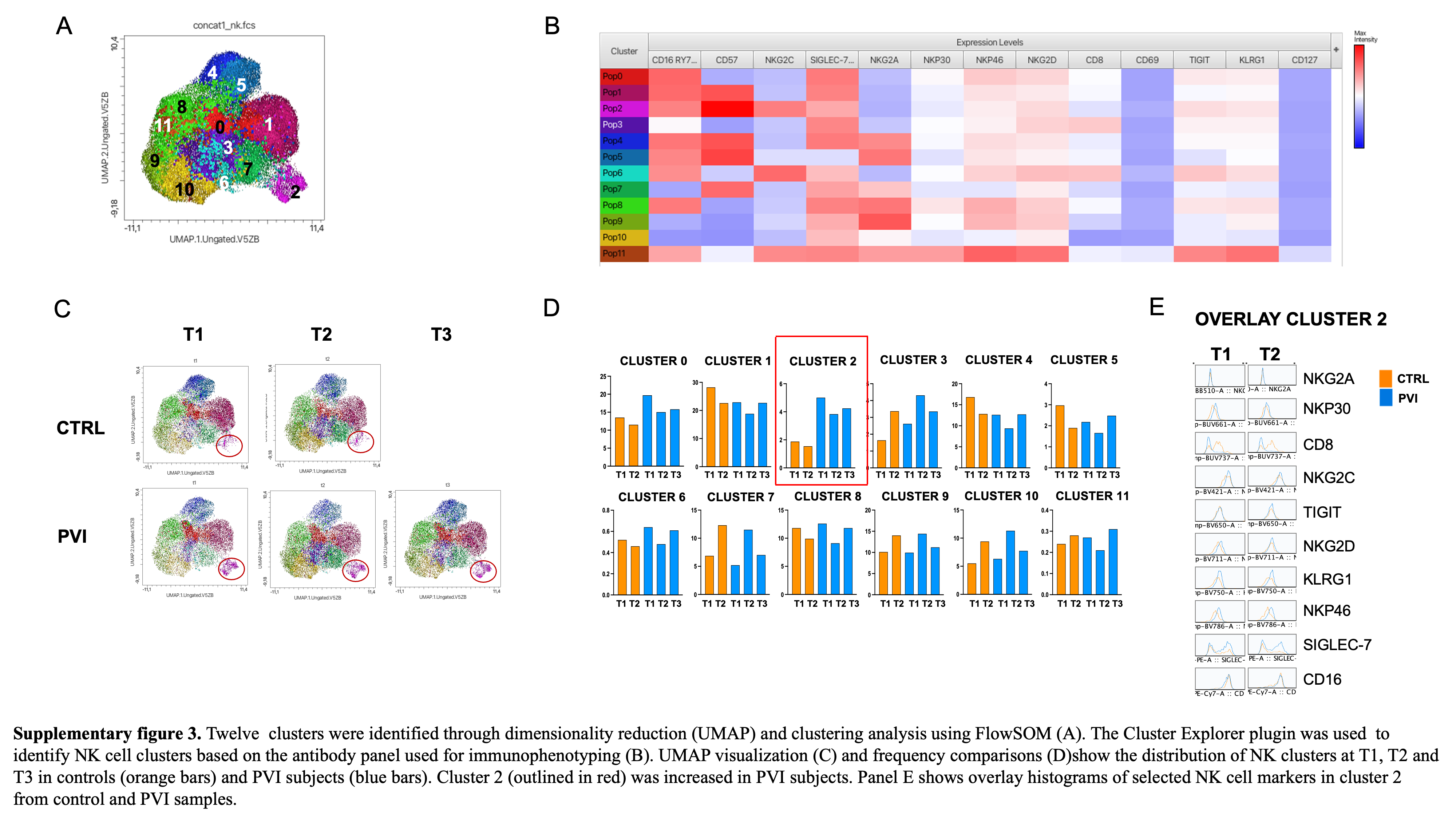

Supplement: Supplementary file 3 [file Image3.tiff]
